# National norms for the obstetric nurses’ and midwives’ health education competence, and its influencing factors: a nationwide cross-sectional study

**DOI:** 10.1186/s12909-024-05249-w

**Published:** 2024-04-09

**Authors:** Jingjing Zou, Jingling Wu, Xiumin Jiang

**Affiliations:** 1 School of Nursing, Fujian University of Traditional Chinese Medicine, Fuzhou, Fujian Province China; 2 Women’s Hospital, School of Medicine, Zhejiang University, Hangzhou, Zhejiang Province China; 3 Fujian Maternity and Child Health Hospital College of Clinical Medicine for Obstetrics & Gynecology and Pediatrics, Fujian Medical University, No.18 Daoshan, Fuzhou, Fujian Province China

**Keywords:** Health education, Competence, Obstetric nurse, Midwifery, Norms, Factors

## Abstract

**Background:**

Strengthening obstetric nurses’ and midwives’ health education competence is the investment and guarantee for the population’s future health. The purpose of study is to establish national norms for their health education competence, and explore possible influencing factors for providing an uniform criterion identifying levels and weaknesses.

**Methods:**

An online questionnaire with a standard process was used to collect data. Three normative models were constructed, and multiple linear regression analysis analyzed possible influencing factors.

**Results:**

The sample respondents (*n* = 3027) represented obstetric nurses and midwives nationally. Three health education competency normative norms (mean, percentile and demarcation norm) were constructed separately. Locations, hospital grade, department, marital status, training times and satisfaction with health education training influenced obstetrical nurses’ and midwives’ health education competence (*P*<0.05).

**Conclusion:**

This study constructed the first national standard for assessing obstetric nurses’ and midwives’ health education competence, providing a scientific reference to evaluate the degree of health education competence directly. These known factors could help clinical and policy managers designate practice improvement measures. In future research, Grade I hospitals should be studied with larger sample sizes, and indicators need to improve to reflect health education’s effect better.

## Background

The World Health Organization has long recommended [1] that pregnant women need more health education, life guidance, and follow-up visits. “Outline of the Healthy China 2030 Plan” [2, 3] also proposed that health services would be strengthened to improve the health of women and children, and it is essential to provide health education covering the prenatal, perinatal and postnatal periods. Comprehensive and practical health education can significantly enhance maternal and infant safety, promote spontaneous delivery, and increase exclusive breastfeeding rates [4]. Authorities such as the International Confederation of Midwives [5] and the American College of Nurse-Midwives [6] emphasise the critical role of midwives and obstetric nurses in providing comprehensive care, assisting in labour and delivey, and managing complications [7]. Obstetric nurses and midwives should possess extensive health knowledge and excellent education competence to ensure women and their families can make informed decisions, and safely manage maternal health and well-being [8].

Strengthening obstetric nurses’ and midwives’ health education competence is the investment and guarantee for the population’s future health [9]. Given its importance, researchers have conducted in-depth discussions on health education quality, goals, strategy and evaluation. However, no study has built a uniform criterion for assessing the performance of obstetric nurses’ and midwives’ health education competency. A norm, a reference standard for the scores obtained using a scale, is usually the average score and standard deviation of many testers. A norm could compare the differences between different groups and assess individual performance [10]. Meanwhile, based on the normal analysis, a more scientific and reliable scale promotion strategy can be formed to popularize and promote relevant scientific theories and methods [11].

The rating scale of health education competence for nurses (RSHECN) was developed and verified its reliability and validity (Tong and Li, 2010). The scale determined that good performance in health education requires nurses to have adequate expertise, sound assessment, planning and implementation and the ability to evaluate the effectiveness of health education, which calcified the connotation of health education competence for nurses and provided a way for evaluation. Therefore, a nationwide cross-sectional survey of multi-stage stratified cluster sampling was conducted to establish norms for RSHECN and explore their influencing factors of health education competence, providing a reference for clinical and policy managers to identify weaknesses and formulate practice improvement plans.

## Method

### Study design

A cross-sectional study of multi-stage stratified cluster sampling was carried out [12, 13]. The nationwide obstetric nurses and midwives were selected as participants from April to May 2021 to establish the mean norm, percentile norm and demarcation norm of RSHEC and explore possible influencing factors of obstetric nurses’ and midwives’ health education competence.

### Participants

The participants were recruited using a stratified multistage cluster sampling method with three steps: (1) Selected representative regions. Three regions (Eastern China, Central China and Western China) were selected, divided by the National Bureau of Statistics of China according to geographic location and economic level. (2) Selected provincial administrative unit (from now it was referred to as the “unit”). The convenient sampling method was used to decide the final units. Seven out of eleven in the eastern region were selected: Tianjin, Hebei, Liaoning, Jiangsu, Zhejiang, Fujian and Guangdong. Four out of eight units in the central region were selected: Shanxi, Heilongjiang, Jiangxi and Hunan. Seven out of twelve units in the western region were selected: Sichuan, Chongqing, Gansu, Qinghai, Xinjiang, Guangxi and Inner Mongolia. (3) Selected included hospitals. The selection of hospitals adopted a convenient sampling method and ensured the diversity of garde I, II and III hospitals as much as possible. After that, with the consent of the hospital nursing department, a whole-group sampling method was used to include all obstetric nurses who met the inclusion criteria in the included hospitals. All active registered obstetrical nurses or midwives who voluntarily participated were included in this study. Moreover, interns, visiting nurses, and nurses who were absent during the survey or could not attend for personal reasons were excluded. The ethical committee of the principal researcher’s hospital approved the study (No 2018 − 206). Before the survey, written consent was obtained from all nursing departments. The questionnaire does not collect the personal information of the participants, and the database can only be accessed by the members of the research group. Participants were informed consent, and the returning online questionnaire was considered consent of participation.

### Data collection

An introduction letter stating the study aim and process was issued to the department of the selected hospital to obtain survey permission. Then the online training was held to conduct a unified training for the hospital responsible person for the project. The standard data collection process was introduced to the responsible person with a standard language, and the contact information of the research group was provided to communicate the problem during the survey. The standard data collection process is the following: (1) Screen potential participants according to inclusion and exclusion criteria; (2) Seek the consent of potential participants. (3) Emphasize anonymity and confidentiality and sign the informed consent; (4) Invite participants to complete the questionnaire. Considering workforce and material resources, this survey adopts electronic questionnaires by the software “Questionnaire Star”, which helps to distribute questionnaires more scientifically in such an extensive national survey. The procedure was set so that each electronic device could only fill in the questionnaire once and submit the questionnaire after completing all items within 30 min. At the end of the survey, 5% of the questionnaires were randomly selected for quality check.

### Measurements

The health education competence assessment questionnaire involves two parts: (1) general information questionnaire: The questionnaire was designed by reviewing relevant literature research and discussing with obstetric nursing experts, which covered the potential factors that might affect the health education competence of obstetrical nurses and midwives, including the type of hospital, age, educational level, current work department, additional training in health education, working years and other basic social demographic information. (2) Rating Scale of Health Education Competence for Obstetric Nurses and Midwives. The scale was used to evaluate the competence of health education of nurses and midwives, which had been authorised by the developer of Tong [14]. The self-evaluation scale includes four dimensions: assessment, plan, implementation and evaluation. Thirty-eight items on a five-point Likert-type scale (1 to 5, “completely disagree” to “completely agree”) and all items are positive. The score ranges from 37 to 185, and higher scores indicate better health education competence. The psychological verification was completed among various departments, including the obstetric nurse and midwife. The scale’s Cronbach α and half-fraction reliability were 0.949 and 0.953, the content validity index was 0.90, and it was verified with good construction validity and distinguish validity [14]. In this sample, 500 questionnaires were randomly selected in proportion to the number of obstetric nurses and midwives for reliability testing, and its Cronbach α was 0.987. Moreover, to facilitate understanding and comparison, the results of this study were analysed using conversion score, and the formula is as follows: conversion score = (original score theoretical minimum score of this aspect) / (theoretical maximum score theoretical minimum score of this aspect) ×100.

### Data analysis

All calculations were performed using IBM SPSS Statistics software (version 26.0). Continuous variables were reported as mean ($\stackrel{-}{\text{x}}$) ± standard (S), and categorical variables were presented as frequencies and proportions. Three types of norms were developed in this study to establish normative values for health education competence among obstetric nurses and midwives. The mean norm was determined using the results of one-way ANOVA to calculate the mean and standard deviation of conversion scores and each dimension score. Percentile norm was established using the percentile method, with 5% percentile intervals, resulting in normative values at the 5th, 25th, 50th, 75th, and 95th percentiles. The demarcation norm was established through the distribution method with different demarcation schemes calculated at a spacing of 0.5 S within the total scale score ($\stackrel{-}{x}$ ± 2.5 S). After that, we performed correlation analysis and selected the scheme with the highest correlation as the demarcation constant for the study [15]. Differences in assessment, plan, implementation, evaluation and conversion scores were analysed using an independent two-sample t-test and one-way analysis of variance, with demographic characteristics as independent variables. Statistically significant variables from the ANOVA analysis were included as independent variables in a stepwise multiple linear regression analysis to evaluate their contributions to conversion scores. In this study, covariance diagnosis of independent variables is based on tolerance (TOL) and variance inflation factor (VIF), and if TOL < 0.1 or VIF ≥ 10, it means that there is serious covariance between independent variables.

## Findings

Three thousand three hundred questionnaires were received, 97 were excluded due to logical self-contradiction of data and abnormal distribution of values, and 3207 questionnaires were available with an effective recovery of 97.18%. All participants were female between eighteen and sixty-four years (33.20 ± 7.51 years). They had one to forty-five working years with an average of (11.00 ± 8.15) years covering the general population for job title, education, department and health education training conditions. Detailed demographic characteristics of the sample are shown in Table 1.

**Table 1:** Demographics characteristics (*n* = 3207)

Variables	Levels	N	%
Hospital grade	Grade III	2102	65.5
	Grade II	1064	33.2
	Grade I	41	1.3
Hospital Type	General Hospital	2332	72.8
	Specialist hospital	136	4.2
	Maternal and Child Health Hospital	701	21.9
	Private Hospitals	35	1.1
Locations	Eastern China	1665	51.9
	Central China	354	11.0
	Western China	1188	37.0
Age (years)	≤ 25	404	12.6
	26–35	1823	56.8
	36–45	717	22.4
	≥ 46	263	8.2
Working years	≤ 5	852	26.6
	6–10	1035	32.3
	11–15	513	16.0
	16–20	356	11.1
	≥ 21	451	14.1
Department	Delivery room	1799	56.1
	Obstetrics ward	1306	40.7
	Obstetrics clinic	102	3.2
Job title	Primary nurse	542	16.9
	Senior nurse	1426	44.5
	Supervisor nurse	943	29.4
	Co-chief nurse	251	7.8
	Professor of nursing	45	1.4
Education level	Technical secondary school	69	2.2
	Professional training college	980	30.6
	Bachelor’s degree or above	2158	67.3
Marital status	Married	2395	74.7
	Unmarried	770	24.0
	Other	42	1.3
Training times of health education	≤ 10	3106	96.9
	11–19	40	1.2
	≥ 20	61	1.9
Satisfaction with health education training	Very satisfied	1454	45.3
	Satisfied	1138	35.5
	Generally satisfied	564	17.6
	Less satisfied	26	0.8
	Very dissatisfied	25	0.8

Mean norms could be established for groups with different characteristics in the tested population. Considering the different economic and medical levels, five categorical mean norms were determined, including grade III hospitals, grade II hospitals, eastern China, central China and western China (Table 2). There is no specification Grade I for hospitals because of the insufficient sample size of primary hospitals (only 41 nurses from Grade I hospitals). The percentile norm was calculated based on scale scores and each dimension score at an interval of 5%, as shown in Table 3. The distribution method was used to establish the demarcation norm, and plan 4, with the highest correlation coefficient (*r* = 0.970), was selected as the final scheme, as shown in Table 4. The final demarcation grade was extremely poor [0, 70.32), poor [70.32, 76.5), medium [76.5, 88.86), good [88.86, 95.04), and excellent [95.04, 100].

**Table 2:** Mean norm for scale score and each dimension score of rating scale of health education competence for obstetric nurses

Variables	East China		Central China		West China	
	Grade III *n* = 1019	Grade II *n* = 616	Grade III *n* = 228	Grade II *n* = 124	Grade III *n* = 855	Grade II *n* = 324
Assessment	83.96 ± 12.74	82.09 ± 12.68	85.05 ± 13.09	82.28 ± 13.47	81.44 ± 12.57	80.20 ± 13.09
Plan	83.14 ± 13.41	81.59 ± 13.20	85.55 ± 13.45	81.22 ± 13.88	80.90 ± 13.04	79.76 ± 14.40
Implementation	84.51 ± 12.94	82.81 ± 12.50	86.70 ± 12.55	83.30 ± 12.77	82.48 ± 12.62	81.39 ± 13.47
Evaluation	84.01 ± 13.36	81.85 ± 12.93	85.51 ± 13.32	82.63 ± 13.21	81.85 ± 12.47	80.89 ± 14.42
Scale	83.97 ± 12.48	82.18 ± 12.17	85.77 ± 12.37	82.44 ± 12.58	81.73 ± 11.94	80.60 ± 12.92

**Table 3:** Percentile norm for scale and each dimension score of rating scale of health education competence for obstetric nurses

Variables	Percentile (%)	Grade III	Grade II	East	Central	West
Scale	5	66.22	64.36	66.22	66.22	64.19
	25	75.00	74.32	75.00	75.00	74.32
	50	79.05	76.35	77.70	81.76	75.68
	75	97.30	95.78	97.30	99.32	93.91
	95	100.00	100.00	100.00	100.00	100.00
Assessment	5	63.64	61.36	63.64	61.36	61.36
	25	75.00	75.00	75.00	75.00	75.00
	50	79.54	77.27	77.27	81.82	75.00
	75	97.73	95.45	97.73	98.30	93.18
	95	100.00	100.00	100.00	100.00	100.00
Plan	5	62.50	59.38	62.50	62.5	59.38
	25	75.00	75.00	75.00	75.00	75.00
	50	75.00	75.00	75.00	78.13	75.00
	75	100.00	96.88	100.00	100.00	93.75
	95	100.00	100.00	100.00	100.00	100.00
Implementation	5	68.75	66.67	68.75	68.75	64.58
	25	75.00	75.00	75.00	75.00	75.00
	50	77.08	75.00	77.08	83.33	75.00
	75	100.00	97.92	100.00	100.00	97.92
	95	100.00	100.00	100.00	100.00	100.00
Evaluation	5	66.67	62.50	66.67	66.67	66.67
	25	75.00	75.00	75.00	75.00	75.00
	50	75.00	75.00	75.00	79.17	75.00
	75	100.00	100.00	100.00	100.00	100.00
	95	100.00	100.00	100.00	100.00	100.00

**Table 4:** Demarcation norm for conversion score of rating scale of health education competence for obstetric nurses and midwife

Plan	Extremely Poor	Poor	Medium	Good	Best	Correlation
1	[0, $\stackrel{-}{x}$−2.5s) [0, 51.78)	$[\stackrel{-}{x}-2.5\text{s}, \stackrel{-}{x}-0.5\text{s}$) [51.78, 76.5)	$[\stackrel{-}{x}-0.5\text{s}, \stackrel{-}{x}+0.5\text{s}$) [76.5, 88.86)	$[\stackrel{-}{x}+0.5\text{s}, \stackrel{-}{x}+2.5\text{s}$) [88.86, 113.58)	[$\stackrel{-}{x}$+2.5s,100] [113.58, 100]	0.939**
2	[0, $\stackrel{-}{x}$−2.0s) [0, 57.96)	$[\stackrel{-}{x}-2.0\text{s}, \stackrel{-}{x}-0.5\text{s}$) [57.96, 76.5)	$[\stackrel{-}{x}-0.5\text{s}, \stackrel{-}{x}+0.5\text{s}$) [76.5, 88.86)	$[\stackrel{-}{x}+0.5\text{s}, \stackrel{-}{x}+2.0\text{s}$) [88.86, 107.4)	[$\stackrel{-}{x}$+2.0s,100] [107.4, 100]	0.948**
3	[0, $\stackrel{-}{x}$−1.5s) [0, 64.14)	$[\stackrel{-}{x}-1.5\text{s}, \stackrel{-}{x}-0.5\text{s}$) [64.14, 76.5)	$[\stackrel{-}{x}-0.5\text{s}, \stackrel{-}{x}+0.5\text{s}$) [76.5, 88.86)	$[\stackrel{-}{x}+0.5\text{s}, \stackrel{-}{x}+1.5\text{s}$) [88.86, 101.22)	[$\stackrel{-}{x}$+1.5s, 100] [101.22, 100]	0.956**
4	[0, $\stackrel{-}{x}$−1.0s) [0, 70.32)	$[\stackrel{-}{x}-1.0\text{s}, \stackrel{-}{x}-0.5\text{s}$) [70.32, 76.5)	$[\stackrel{-}{x}-0.5\text{s}, \stackrel{-}{x}+0.5\text{s}$) [76.5, 88.86)	$[\stackrel{-}{x}+0.5\text{s}, \stackrel{-}{x}+1.0\text{s}$) [88.86, 95.04)	[$\stackrel{-}{x}$+1.0s, 100] [95.04, 100]	0.970**
5	[0, $\stackrel{-}{x}$−2.5s) [0, 51.78)	$[\stackrel{-}{x}-2.5\text{s}, \stackrel{-}{x}-1.0\text{s}$) [51.78, 70.32)	$[\stackrel{-}{x}-1.0\text{s}, \stackrel{-}{x}+1.0\text{s}$) [70.32, 95.04)	$[\stackrel{-}{x}+1.0\text{s}, \stackrel{-}{x}+2.5\text{s}$) [95.04, 113.58)	[$\stackrel{-}{x}$+2.5s, 100] [113.58, 100]	0.898**
6	[0, $\stackrel{-}{x}$−2.0s) [0, 57.96)	$[\stackrel{-}{x}-2.0\text{s}, \stackrel{-}{x}-1.0\text{s}$) [57.96, 70.32)	$[\stackrel{-}{x}-1.0\text{s}, \stackrel{-}{x}+1.0\text{s}$) [70.32, 95.04)	$[\stackrel{-}{x}+1.0\text{s}, \stackrel{-}{x}+2.0\text{s}$) [95.04, 107.4)	[$\stackrel{-}{x}$+2.0s, 100] [107.4, 100]	0.896**
7	[0, $\stackrel{-}{x}$−1.5s) [0, 64.14)	$[\stackrel{-}{x}-1.5\text{s}, \stackrel{-}{x}-1.0\text{s}$) [64.14, 70.32)	$[\stackrel{-}{x}-1.0\text{s}, \stackrel{-}{x}+1.0\text{s}$) [70.32, 95.04)	$[\stackrel{-}{x}+1.0\text{s}, \stackrel{-}{x}+1.5\text{s}$) [95.04, 101.22)	[$\stackrel{-}{x}$+1.5s, 100] [101.22, 100]	0.881**
8	[0, $\stackrel{-}{x}$−2.5s) [0, 51.78)	$[\stackrel{-}{x}-2.5\text{s}, \stackrel{-}{x}-1.5\text{s}$) [51.78, 64.14)	$[\stackrel{-}{x}-1.5\text{s}, \stackrel{-}{x}+1.5\text{s}$) [64.14, 101.22)	$[\stackrel{-}{x}+1.5\text{s}, \stackrel{-}{x}+2.5\text{s}$) [101.22, 113.58)	[$\stackrel{-}{x}$+2.5s, 100] [113.58, 100]	0.414**
9	[0, $\stackrel{-}{x}$−2.0s) [0, 57.96)	$[\stackrel{-}{x}-2.0\text{s}, \stackrel{-}{x}-1.5\text{s}$) [57.96, 64.14)	$[\stackrel{-}{x}-1.5\text{s}, \stackrel{-}{x}+1.5\text{s}$) [64.14, 101.22)	$[\stackrel{-}{x}+1.5\text{s}, \stackrel{-}{x}+2.0\text{s}$) [101.22, 107.4)	[$\stackrel{-}{x}$+2.0s, 100] [107.4, 100]	0.413**
10	[0, $\stackrel{-}{x}$−2.5s) [0, 51.78)	$[\stackrel{-}{x}-2.5\text{s}, \stackrel{-}{x}-2.0\text{s}$) [51.78, 57.96)	$[\stackrel{-}{x}-2.0\text{s}, \stackrel{-}{x}+2.0\text{s}$) [57.96, 107.4)	$[\stackrel{-}{x}+2.0\text{s}, \stackrel{-}{x}+2.5\text{s}$) [107.4, 113.58	[$\stackrel{-}{x}$+2.5s, 100] [113.58, 100]	0.323**

^**^P < 0.001

The results of one-way ANOVA showed statistically significant differences (*P* < 0.05) in the health education competency conversion scores comparing hospital type, hospital grade, department, locations, marital status, satisfaction with health education training, and training times of health education. The multiple linear regression analysis showed that hospital grade (*P* = 0.002), locations (*P* = 0.032), department (*P* = 0.001), marital status(*P* = 0.003), satisfaction with health education training (*P* < 0.001), and training times of health education (*P* = 0.006) were independent influencing factors of obstetric nurses’ and midwives’ health education competency scores. In this study, the TOL values were 0.956–0.993 and VIF values were 1.007–1.046, which cannot be considered as the existence of multiple covariance between independent variables, and all independent variables can be analysed by multiple regression.

## Discussion

This study established the first national norms for obstetric nurses’ and midwives’ health education competency and explored possible influencing factors. The mean norm can be used to determine whether obstetric nurses’ and midwives’ health education competency is within the reference range [15]. The result showed that the health education competency was highest in Central areas, followed by Eastern areas, and the lowest in Western areas. The central and east areas have superior medical resources, attracting more medical and nursing talents, while the western region has more mountainous areas with less developed medical resources. Central region scores higher than East region, probably because Central region contains fewer cities. The sample size of this survey is smaller, which makes its average score higher. The mean norm describes the overall level, and the percentile norm was formed to compare the individual score within the corresponding percentile norm to identify individual positions in the group [16, 17]. The higher the score, the higher the percentile norm position, which means the health education competency level is better. The result of showed that the best division scheme was extremely poor [0, 70.32), poor [70.32, 76.5), medium [76.5, 88.86), good [88.86, 95.04) and excellent [95.04, 100], which make the scores for different indicators can be compared easily, reducing the difficulty of interpreting and comparing data, while also allowing for a more intuitive and accurate assessment of individual performance.

In this study, the mean scale score was (82.68 ± 12.36), which is intermediate compared to the norm [18, 19]. The conversion scores from highest to lowest were implementation, evaluation, assessment and planning, consistent with clinical practice. In the clinical environment, each pregnant woman has different educational needs. However, nurses, as mainly part of implementer of health education, only teach fixed content but do not individualise health education on a case-by-case basis. Although there are often many research materials, such as guidelines, to guide obstetric nurses and midwives on what to do, they often copy and use indoctrination again, lacking individualised assessment of pregnant women [20]. Thus, the result prompts us to form a practical health education model in line with national conditions, strengthing the status of evaluation, assessment and planning to provide individualised health education and play the role of health education better.

The study identified that locations, hospital grade, department, marital status, satisfaction with health education training and training times were influencing factors for obstetric nurses’ and midwives’ health education competence. Among different locations, the disparity in medical conditions may lead to managers with different perceptions on the role of nurses’ and midwives’ in health education. Within health care teams, obstetric nurses and midwives are vital health education providers throughout the pregnancy and delivery. The government could introduce more policies and supportive steps to improve the attention of hospitals in underdeveloped areas to the health education capacity of nurses.

The score of tertiary hospitals was higher than secondary, and the possible reason is that tertiary hospitals absorbs higher qualified nursing talents [21, 22], and they have more robust medical resources, research and teaching capabilities to provide more professional training and education and are more excellent regarding professional qualifications and skills [23]. Meanwhile, the regression analysis showed that the times and satisfaction of health education training were influencing factors. Long-term participation in health education training could enhance the professional confidence, stability and self-confidence of obstetrical nurses and midwives [23, 24]. Satisfactory training can encourage applying knowledge and skills in practical work, promoting health education competency and work continuity [25]. Each training is a process of knowledge accumulation, and the increasing knowledge reserve in reproductive health, prenatal, intrapartum and postpartum care can better guide maternal health management and improve the life quality of birthing mothers and their infants [26, 27]. Therefore, for hospitals managers, the organization of comprehensive, professional and satisfactory health education knowledge training should be regarded as an important part of management, especially for grassroots hospitals.

Another interesting result is that the health education competence of married and fertile nurses was better, who can better feel the actual needs of pregnant women and combine their own experience to provide more detailed and thoughtful health education in dealing with various real situations [28, 29]. Future research can explore more health education methods from the perspective of maternity, so as to help unmarried and infertile nurses and midwives. Our result also showed that midwives scored lower than obstetric nurses, which may be due to the different work nature. Generally, obstetric nurses provide health education in the ward, while midwives in the delivery room. The unique physiological conditions for childbirth can make it challenging to provide health education. And the demand for health education after delivery is more significant, as the mother and her family require more information about puerperal rehabilitation and neonatal care. When providing health education, midwives and obstetric nurses could promote strengths and avoid weaknesses. Obstetric nurses can provide comprehensive health education for mothers and their families after delivery, and midwives can try to move forward their own health education opportunities and provide health education in midwives’ outpatient clinics.

A normative standardised reference will serve as a reference to help obstetric nurses and midwives identify strengths and weaknesses in health education competence and help management establish a more reasonable nursing echelon for enhancing maternal health [30, 31]. The nationwide cross-sectional survey could help clinical and policy managers understand the current health education situation and formulate corresponding management plans for practice improvement [32, 33]. Although the results reported here are of interest, it is necessary to acknowledge certain limitations of the study. Firstly, due to time and human constraints, the small sample size of the Grade I hospitals in this study affected the completeness of the norm. Also, the convenience sampling method used for hospital selection might introduce bias, as it does not ensure a randomized and comprehensive representation of all hospital grades, particularly Grade I hospitals. Future studies should aim for a more extensive and diverse sample, including a better representation of all hospital grades. Secondly, the study is limited to a specific time frame, which may not adequately represent changes over time. A longitudinal approach could offer insights into how health education competence evolves over time and its long-term impact on patient care and outcomes. Thirdly, the scale is a self-assessment scale, which is subjective in evaluating health education competence and lacks objective evaluation indicators. Obstetric nurses and midwives with higher scores indicate a certain level of health education competence. However, the effect of health education is not reflected by objective indicators, which need to be improved in future studies. Finally, implementing and evaluating training interventions could provide practical insights into effective strategies for improving health education competence among obstetric nurses and midwives.

## Conclusion

A nationwide cross-sectional study of multi-stage stratified cluster sampling was conducted to establish the first national norms for obstetric nurses’ and midwives’ health education competency. Locations, hospital grade, department, marital status, satisfaction with health education training and training times were independent influencing factors for obstetric nurses’ and midwives’ health education competence. The study provides a valid way to assess obstetric nurses’ and midwives’ health education competency comprehensively and comparatively. It helps practitioners make more informed choices when developing relevant programs or decisions. In future research, Grade I hospitals should be studied with larger sample sizes, and indicators need to improve to reflect health education’s effect better.

## Data Availability

The datasets used and/or analysed during the current study are available from the corresponding author on reasonable request.
